# Gene Regulatory Changes Associated With Phenological Transitions in an Ecologically Significant Tree Species

**DOI:** 10.1002/pei3.70078

**Published:** 2025-08-04

**Authors:** Theresa Caso‐McHugh, David L. Des Marais, Miranda Oseguera, Meghan Blumstein

**Affiliations:** ^1^ Civil and Environmental Engineering Massachusetts Institute of Technology Cambridge Massachusetts USA; ^2^ Department of Environmental Sciences University of Virginia Charlottesville Virginia USA; ^3^ School of Architecture University of Virginia Charlottesville Virginia USA

**Keywords:** climate change, genotype, growth chamber, phenology, photoperiod, *Quercus rubra*, red oak, temperature

## Abstract

Climate change is driving earlier spring leaf‐out across temperate regions, but the genetic mechanisms and environmental interactions underlying this variability are poorly understood. We conducted a controlled growth chamber experiment using excised northern red oak ( *Quercus rubra* ) branches, testing the influence of temperature and photoperiod on leaf development. Two genotypes of red oak were exposed to four different warming and daylength treatments, and gene expression was analyzed across stages of bud development. Results revealed significant phenotypic differences between genotypes and across treatments, confirming that leaf‐out timing is both genetically determined and environmentally responsive. Our analysis identified several key genes involved in dormancy break and photoperiod sensitivity, including orthologs to genes identified in *Populus* species, suggesting conserved pathways across tree species. These genes were differentially expressed in response to environmental factors, highlighting the polygenic nature of phenological timing. Notably, modules associated with temperature and photoperiod showed overlap with dormancy break pathways, indicating shared regulatory networks. This study provides a foundational dataset for understanding phenology in red oak and offers insights into how genetic and environmental factors shape leaf development in temperate trees, setting the stage for further functional genomic research.

## Graphical abstract

Climate change is driving earlier spring leaf‐out across temperate regions, but the genetic mechanisms and environmental interactions underlying this variability are poorly understood. We conducted a controlled growth chamber experiment using excised northern red oak (*Quercus rubra*) branches, testing the influence of temperature and photoperiod on leaf development and gene expression. Our analysis identified several key genes involved in dormancy break and photoperiod sensitivity, including orthologs to genes identified in Populus species, suggesting conserved pathways across tree species. This study provides a foundational dataset for understanding phenology in red oak and offers insights into how genetic and environmental factors shape leaf development in temperate trees, setting the stage for further functional genomic research.

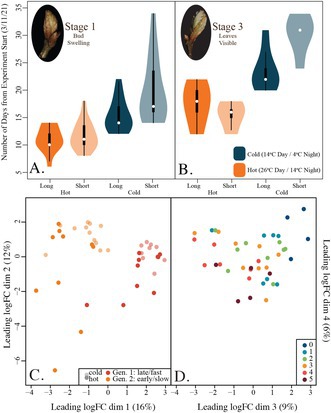

## 1 Introduction

Anthropogenic climate change has driven temperate trees to leaf out earlier in spring. The timing of leaf out in northeastern North America has advanced by 5–10 days since the 1970s (Aitken et al. 2008; Azeez et al. 2021), and the average annual growing season in Europe has increased by at least 10 days since the 1960s (Basler and Körner 2012). Underlying these average shifts is substantial variation in phenological timing measured across for example, (Blumstein et al. 2024) and within species for example, (Bolger et al. 2014; Caffarra and Donnelly 2011). This variation is driven by a plant's genetic background (G), the environmental conditions in which it lives (E), and the interaction between the two (GxE) (Cavender‐Bares et al. 2015).

Decades of observations in wild populations and in experimental manipulations have concluded that forcing (warming in spring), photoperiod (daylength), and chilling (length of dormancy) are the main environmental drivers of variation in spring leaf development timing (Blumstein et al. 2024; Chuine 2000; Cleland et al. 2007; Derory et al. 2006). Higher spring temperatures almost universally lead to earlier leaf‐out. Conversely, shorter and/or warmer dormancy periods may lead to delayed leaf development (Chuine 2000; Derory et al. 2010). Finally, longer days lead to earlier leaf‐out (Donnelly et al. 2011). However, how these cues interact and the relative importance of each in driving leaf‐out timing is poorly understood (Evans et al. 2014; Finzi et al. 2020; Flynn and Wolkovich 2018) and likely differs across species (Blumstein et al. 2024; Franklin and Whitelam 2007).

Variation in leaf‐out timing has also been demonstrated to have a strong genetic basis; it is highly heritable and locally adapted. For example, broad‐sense heritability (H2) of leaf‐out ranges from 0.43–0.72 in *Salix* (Heide 1993), 0.48–0.80 in *Populus* (Howe et al. 2000; Kapoor et al. 2023), and 0.15–0.87 in *Quercus* (Keller et al. 2012). This genetic variation has been shown to be locally adapted to climatic conditions across tree populations (Bolger et al. 2014; Keller et al. 2011; Kim et al. 2002, 2011; Kreyling et al. 2014). While the genetic basis of leaf‐out is likely highly polygenic in structure (Kuznetsova et al. 2017), several large‐effect genes have been identified to be involved with bud burst in *Populus tremula × Populus alba* hybrid (Langfelder and Horvath 2008; Laube et al. 2014; Li and Dewey 2011). The pathway involves low temperatures positively regulating the gene EBB1, which then negatively regulates the gene SVL. SVL forms a positive feedback loop with the hormone ABA, and the loop negatively regulates EBB3. EBB3 is a positive regulator of bud break by directly positively regulating Cyclin D3 (Langfelder and Horvath 2008).

Despite the progress made in the model cross *Populus tremula × Populus alba*, the overall genetic architecture and molecular mechanisms involved in the process of leaf‐out are still poorly understood in nonmodel species (Love et al. 2014; Martin 2011; McKown, Guy, Klapste, et al. 2014). Furthermore, the genes associated with phenological plasticity are even less clear. Here, we aim to examine gene expression across phenological phases and environmental drivers in a controlled growth chamber experiment using the ecologically important species northern red oak ( *Quercus rubra* ); hereafter referred to as “red oak.”

Red oak is an ideal species for this investigation. It is economically and ecologically important to eastern forests (McKown, Guy, Klapste, et al. 2014) and is a large driver of the eastern carbon sink (McKown, Guy, Quamme, et al. 2014). Additionally, red oaks are a tractable genomic system; they are diploid (2 *N* = 24) with a genome size of approximately 750 MBp and a reference genome recently made available (McKown, Klapste, Guy, et al. 2014). In this study, we conducted a controlled growth chamber experiment containing branch cuttings from two phenologically distinct red oak individuals. These cuttings were placed in a factorial array of four warming by daylength treatments. We scored the phenological progression of these cuttings over a simulated spring and collected bud tissue for transcriptomic analysis across stages and treatments. In particular, we sought to test the following questions:

1. How do temperature and photoperiod jointly influence the phenotype of leaf‐out timing in red oak?
2. What genes are involved in dormancy break? Does red oak use orthologous genes to those previously identified in *Populus* to be important for dormancy break? If so, what are the temporal and/or environmentally dependent expression patterns of these orthologs?
3. What genes are involved in plasticity to photoperiod and temperature? As in dormancy break, are there orthologous genes annotated in previous studies involved in these responses?

## 2 Methods

### 2.1 Experimental Setup

In March 2021, 32 *Quercus rubra* tree cuttings were collected in Cambridge, MA from each of two separate canopy trees. Branches were cut approximately 45–50 cm from the tip of the branch with pruning shears. Cuttings were placed on ice and transported to the lab, where 4 cm of the end of each branch was cut underwater and all buds, except the apical buds, were removed. The branches were then placed into Erlenmeyer flasks containing 400 mL of tap water. Each flask contained three randomly assigned samples and the flasks were randomly assigned a place in a 4 × 2 grid within the chamber for a total of 16 samples per treatment.

**FIGURE 1:**
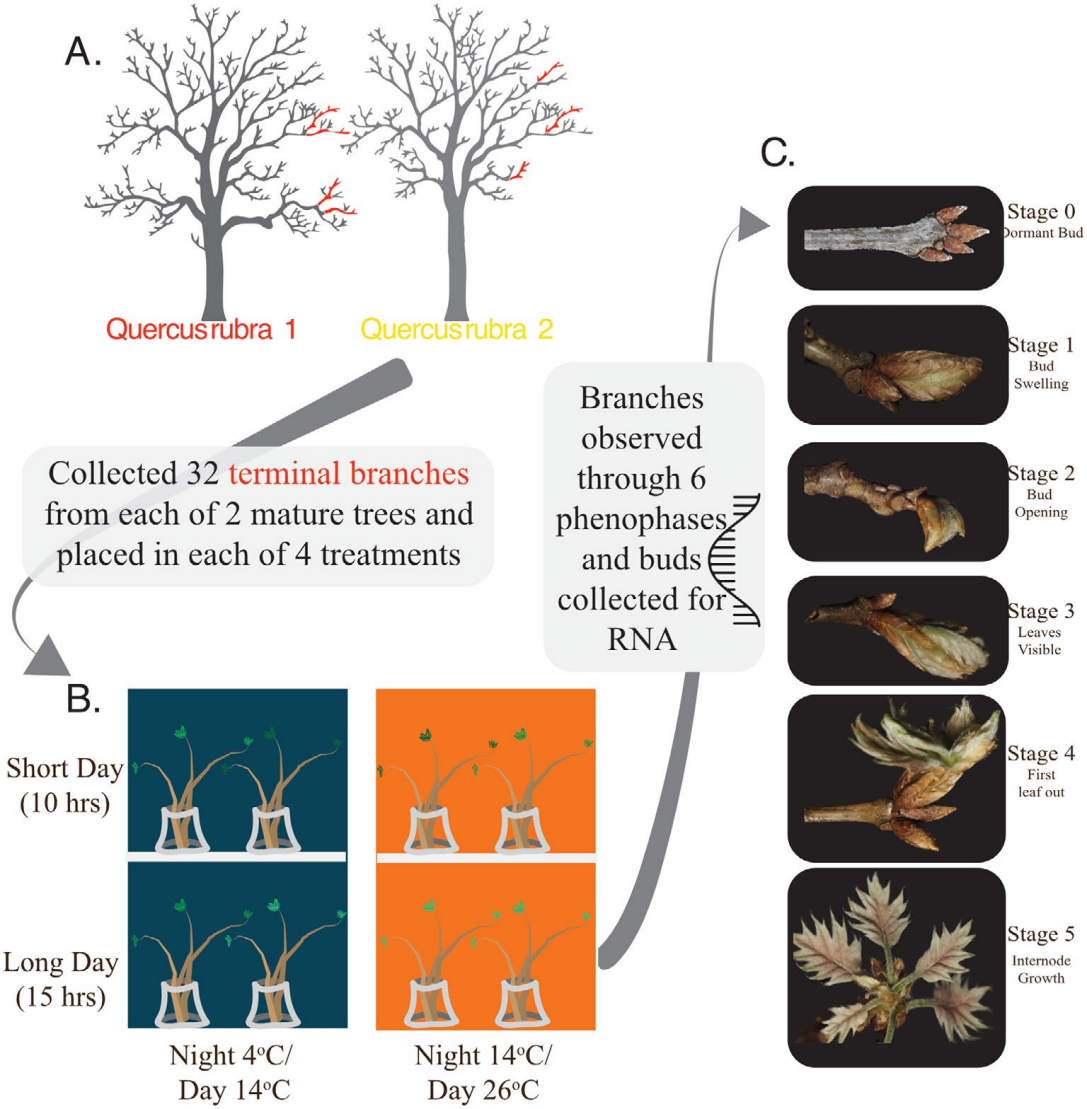
A graphic representing our experimental design. We collected branch cuttings from (A) two red oak individuals with differing leaf‐out timing, then placed them in one of four (B) experimental treatments, and (C) observed each cutting as it advanced through early leaf‐development phenophases, collecting RNA at each new phase.

Flasks were then placed into one of four treatments contained in two BioChambers Model FXC‐9 growth chambers. Each chamber has a shelf with lighting on the underside, dividing the chamber in half horizontally. This setup allowed us to maintain identical temperatures for both shelves but differing light regimes above and below the shelf. One chamber was a “cold” treatment with a temperature of 14°C during the day and 4°C at night. The other chamber was a “hot” treatment with a day temperature of 26°C and a night temperature of 14°C (Figure 1B). These two temperature treatment regimens also had two corresponding photoperiod treatments, a daylength of either 10 (top shelf) or 15 (bottom shelf) hours. All treatments, lights, and day‐warming began each day starting at 6:30 AM.

Phenophase stage observations and the collection of corresponding bud tissue samples were conducted at solar noon every other day. However, during a period in the middle of the study, the quantity and speed of change of bud development were such that daily observations were necessary. For each observation, we conducted inspections of each branch's apical bud to determine bud progression in development (Figure 1C). When one of the three individuals in a genotype progressed to a new stage for the first time (e.g., the first twig that reached stage 2 for *Q. rubra* 1 in the cold/short treatment), this bud was immediately collected into a 2 mL tube and flash frozen in liquid nitrogen before being transferred to a −70°C freezer for storage. Every 2 weeks, water in each flask was replaced with fresh tap water, the bottom 2 cm of each branch was snipped off underwater, and the location of each flask in the growth chamber was randomly shuffled to limit potential chamber effects.

### 2.2 Phenotypes

**FIGURE 2:**
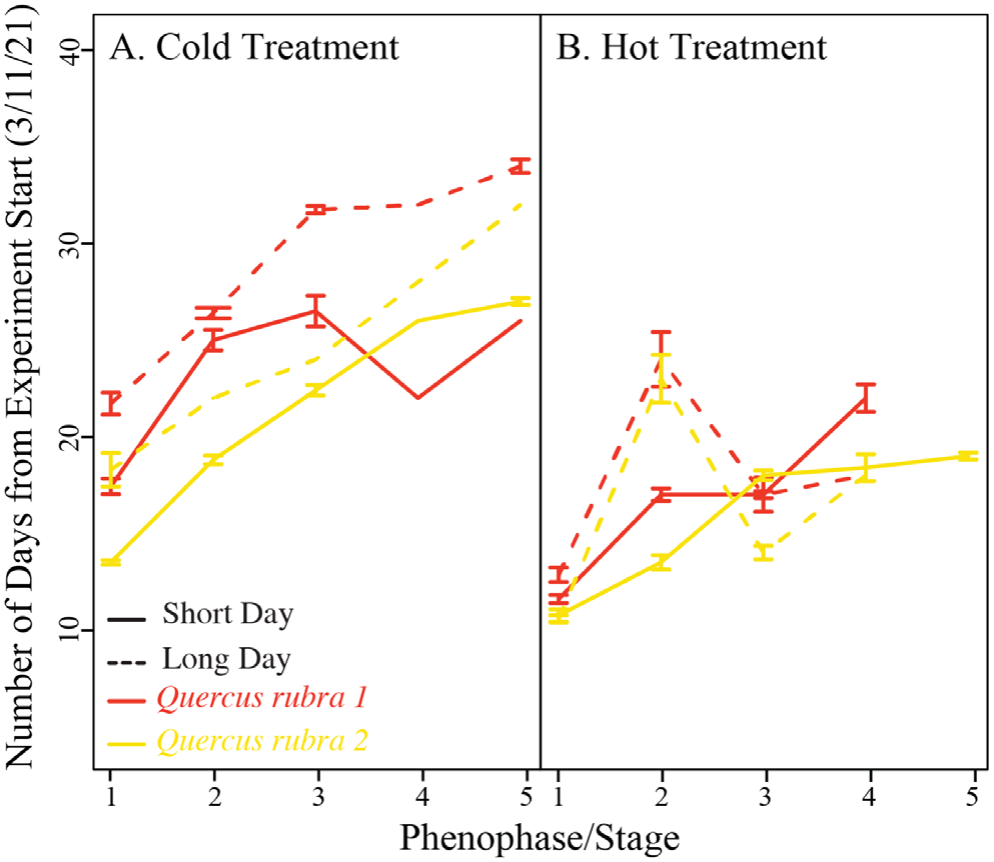
Warmer temperatures and longer days lead to earlier phenological advancement. Data shown for each genotype in the (A) cold (14°C day/4°C night) and (B) hot (26°C day/14°C night) treatments, for both the short day (solid line, 10 h) and long day (dashed line, 15 h) treatments. Error bars represent standard error across samples. As we destructively harvested, the N drops by stages 4 and 5, such that it is not always possible to calculate standard error.

**FIGURE 3:**
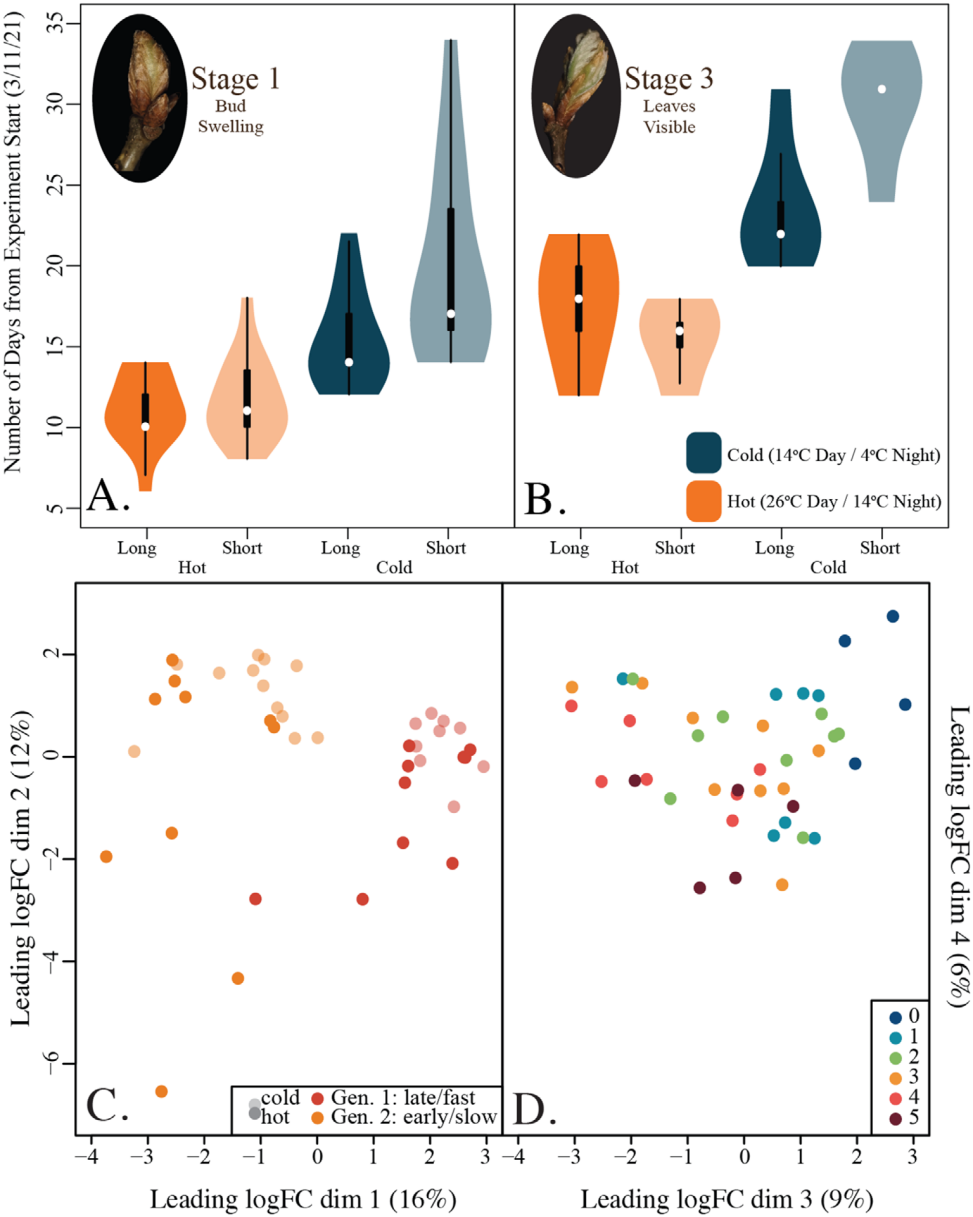
A summary of our phenotypic and RNA results, demonstrating the influence of genotype, photoperiod, and temperature on leaf out on phenotypes and subsequent transcription. Leaf development phenotypes at (A) stage 1 and (B) stage 3. MDS clustering plots of transcriptomic data, showing (C) clustering by genotypes and temperature treatment across dimension 1 & 2 and (D) clustering across stages of phenophase development in dimensions 3 & 4.

To examine differences in leaf‐out phenotypes, we converted data to days from the start of the experiment (3/11/2021) so we could compare the number of days to each stage of leaf development across trees and treatments. We used the package *lmertest* v3.1–3 (Meger et al. 2024) in R to fit mixed models of how the interaction between photoperiod and temperature influences time to each phenological stage:

$$\left(1\right)\;Y_{\mathrm{iG}}=\beta_{\text{Photoperiod}}+\beta_{\text{Temperature}}+\alpha_{G}+\varepsilon_{\mathrm{iG}}$$

where i is the individual sample, G is the genotype from which it came, and Y is the time to the phenological stage. We then focused our analysis on biologically significant stages 1 and 3 (budburst/leaf‐out; Figures 2 and 3; Table 1), although results were similar across all five stages.

**TABLE 1:** Temperature, photoperiod, and the interaction between the two all significantly alter phenology phenotypes. Results are from a linear mixed model run separately from stage 1 and stage 3, with individual genotype fitted as the random effect. We calculated broad‐sense heritability (H^2^) using the random‐effect and residual variance.

		Stage 1		Stage 3		
Fixed Effects	Driver	m (std error)	*p*	m (std error)	*p*	
	Photoperiod (Long)	−4.6 (1.3) days	< 0.001	−5.4 (2.0) days	0.02	
	Temperature (Hot)	−8.3 (1.3) days	< 0.001	−14.0 (2.1) days	< 0.001	
	Interaction (Photo × Temp)	4.2 (1.9) days	0.03	7.8 (3.0) days	0.02	
Random Effects	Variance type	Variance	*H* ^2^	Variance	*H* ^2^	
	Individual	3.1	0.20	3.7	0.26	
	Residual	12.3		10.8		

### 2.3 Transcriptomic

We extracted RNA from tissue samples utilizing the Sigma Spectrum Plant Total RNA kit. Across treatments (4), phenology stages (6), and genotypes (2), we anticipated collecting 48 samples. We missed a collection in one instance due to a quick transition between stages overnight. In the hotter treatment, almost none of the genotypes reached stage 5 in either daylength (¼ lived to the end). In the colder treatment, this only happened to one genotype in the short day treatment (¾ lived to the end). Thus, we ended up with 42 RNA samples.

Sample quality control, next generation library generation, and sequencing were completed by the MIT BioMicro Core Facility with high throughput NEB Ultra II Directional RNA preparations with poly (A) selection to capture mRNA from total RNA. These libraries were sequenced on a NovaSeq6000 for full RNA. Transcripts were aligned to the *Quercus rubra* reference genome v 2.1; (McKown, Klapste, Guy, et al. 2014), which was first converted to a transcriptome using the call “prepare‐reference” from the software RSEM‐1.3.3 (Menzel and Fabian 1999). Reads were first trimmed using the software Trimmoatic‐0.39 (Mimura and Aitken 2010), and then adaptors were cut using the software cutadapt (Paik and Huq 2019). Cleaned paired‐end sequences were then aligned to the oak transcriptome and counted using “calculate‐expression” in RSEM. Finally, reads were combined into a count matrix via the package *tximport* v.1.34.0 in R (v4.2.3) to import the ‘genes. results’ files and format (Richardson et al. 2009). The matrix was filtered to remove genes where the length was 0 or genes for which there were no reads for any samples. Genes with very low variance in reads (standard deviation across all samples < 2.5) as defined as the lowest 10% of genes were also removed. The resultant matrix was then converted into DESeq format using the package *DESeq2* v.1.46.0 (Ritchie et al. 2015).

**FIGURE 4:**
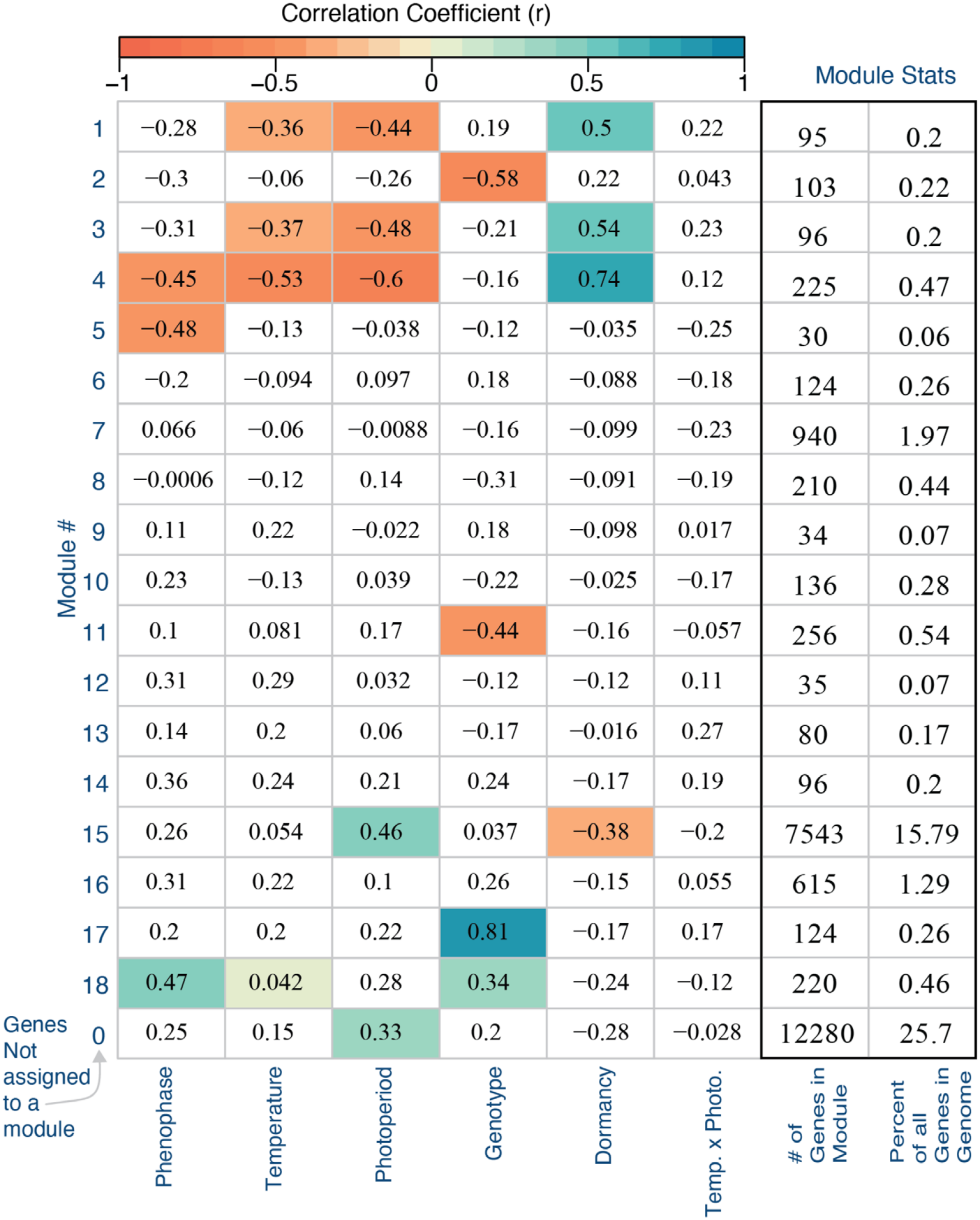
A heatmap summarizing the transcript clustering results and how they correlate with our experimental factors. Only significant interactions (*p* > = 0.05) are colored, with the correlation coefficient (range −1 to 1) indicated in text. Dormancy here is defined as transcriptional differences between stages 0 and the combined stages 1–5. Each of the 18 modules defined varied in the number of genes included, as given by the bolded section of table on the far right, as well as what percentage of total genes in the red oak genome that value represents.

In total, 27,039 unique genes were identified across our samples, which represent 56% of the 47,780 estimated protein‐coding transcripts in *Q. rubra* . Using our expression set, we first examined expression patterns using plotMDS from the limma package *v.3.60.6* Figure 3C,D; (Savage and Chuine 2021). We then used the package WGCNA (*v.1.73*) to conduct a co‐expression network analysis, organizing the gene expression data into clusters (Schwartz et al. 2006). For this analysis, we set the minimum number of genes per cluster to 5, the cut height to 0.995, the merge height to 0.3 (i.e., merge module with 0.7 correlation or more) and found the optimal soft power threshold to be 24. We then ran the clustering algorithm and defined clusters using the dynamic cut height approach. Next, we correlated our module's eigengenes with our various treatments, generating a heatmap summary of our data (Figure 4).

**FIGURE 5:**
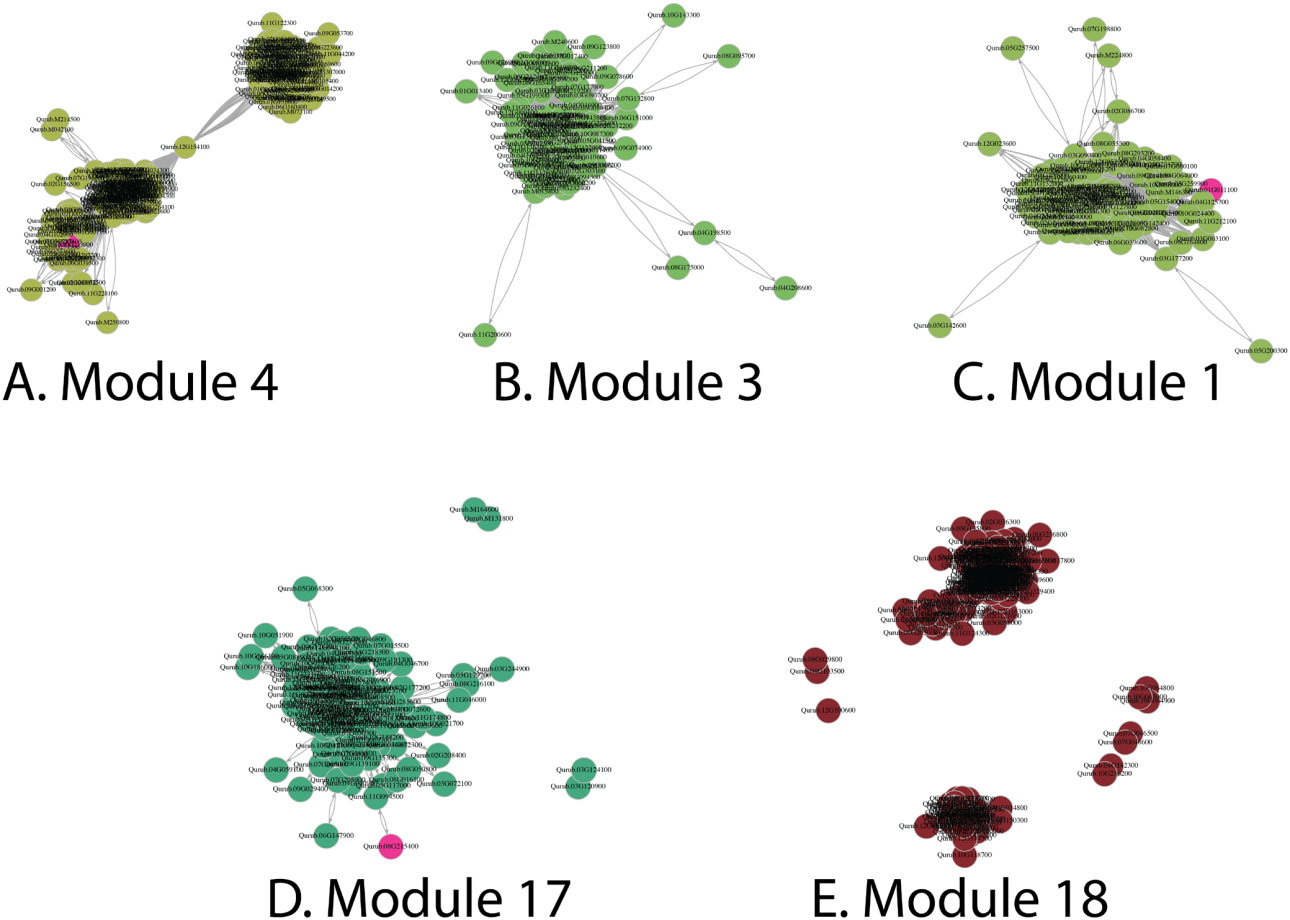
Graphical representations of select transcript modules identified in our study (A–E). Hot pink highlighted genes are leaf‐out related orthologs from other species (Table S1).

We focus, here, on modules most strongly associated with our measured phenologies. First, we visualized these modules using igraph *v.1.5.0* in R (Figure 5). We then created a database of genes associated with spring phenology, as annotated in other tree species in prior studies (Langfelder and Horvath 2008; Scotti‐Saintagne et al. 2004; Singh et al. 2018), and identified their orthologs in the *Q. rubra* genome. To find orthologs, we used the Joint Genome Institute's site Phytozome 13 (https://phytozome‐next.jgi.doe.gov/blast‐search) to identify sequences of the candidate genes, then blast searched for orthologs in the *Q. rubra* genome. The e‐value for matches ranged from 0 to 1.93e‐06, where the closer to 0, the closer to a perfect match between the two gene sequences; based on these criteria, we identified matches for 96 of 100 gene candidates (Table S1). We then queried each module that was associated with a phenology trait for orthologs and calculated the centrality of the candidate gene in the co‐expression network. Unfortunately, only 37% of genes in the red oak genome had gene ontology (GO) annotations, making it infeasible to examine gene functional enrichment terms in an unbiased manner.

**FIGURE 6:**
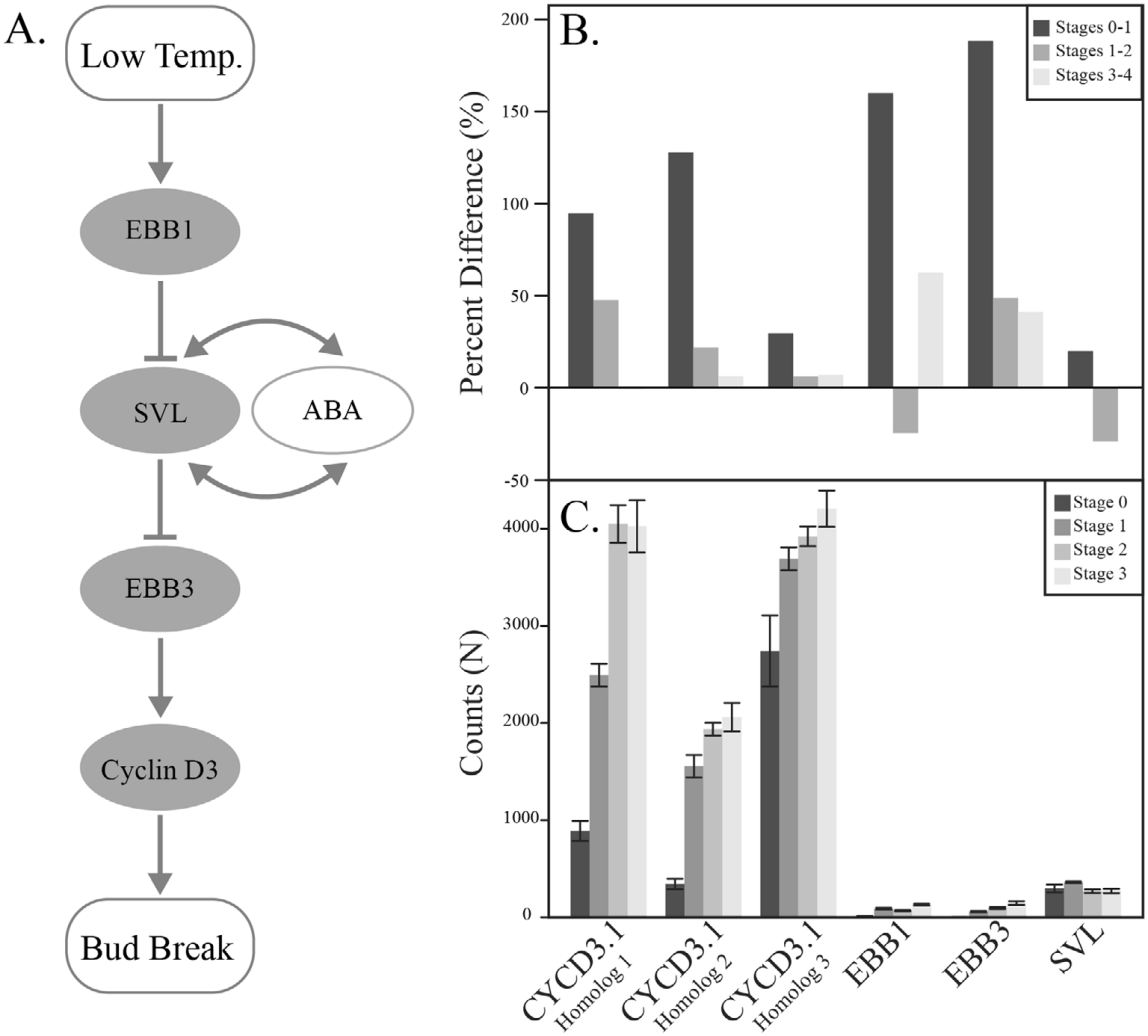
Expression changes in orthologs to genes central to early leaf‐out mutants. The proposed pathway (A) of bud break in a *Populus tremula × Populus alba* hybrid, modified from (Azeez et al. 2021). (B) How expression has changed across the first four stages of leaf‐out (0–3) in our study for those orthologous genes, given as both raw counts and percent differences. Error bars represent standard deviation.

After our broad examination of expression, we looked closely at expression changes in orthologs to genes central to early leaf‐out mutants (Langfelder and Horvath 2008; Li and Dewey 2011) (Figure 6).

## 3 Results

### 3.1 How Do Temperature and Photoperiod Influence the Phenotype of Leaf‐Out Timing in Red Oak?

Warming significantly advanced leaf development timing in both red oak genotypes (Figures 2 and 3; Table 1). In the warmer treatment, time to stage 1 advanced an average of 6 days over the colder treatment, and an average of 10 days by stage 3. Longer photoperiods also resulted in significant advances in leaf‐out (Figure 2, Table 1). By stage 1, longer photoperiods resulted in an average of 2.4 days advance in leaf out and 2.5 days by stage 3 across temperatures and genotypes. We also found a significant interaction between photoperiod and temperature, where colder treatments advanced their leaf‐out timing significantly more under the longer photoperiod treatments than the warmer treatments (Figures 2 and 3A,B, Table 1). MDS clustering of the gene expression data found that these phenotypic differences were largely reflected in the plants' transcriptomes, as well (Figure 3C,D). The first two dimensions of the MDS plot show a divide between genotype (Dim 1) and temperature treatment (Dim 2) (Figure 3C). Dimensions 3 and 4 of the MDS plots reflect the progression of phenological stages from dormancy to fully leafed out (Figure 3D).

### 3.2 What Genes Are Involved in Dormancy Break? Do Red Oak Have Orthologous Genes Previously Identified in *Populus* to Be Important for Dormancy Break? If So, What Are the Expression Patterns of These Orthologs?

Dormancy break—defined here as the phenological progression from stage 0 to stage 1—was significantly associated with four modules (Figure 4). The contrast was most strongly associated with module 4 (Figure 5). Orthologs for a gene in this co‐expression network have previously been identified using GWAS in *Populus trichocarpa* (Table S1). Modules 1 and 3 were also highly associated with dormancy break (Figures 4 and 5). Module 1 contains 2 orthologs of genes identified in a GWAS of budflush as well as an ortholog of a photoreceptor (PIF31) identified in *Populus balsamirifera*, which was originally identified as an ortholog in *Arabidopsis thaliana* (Table S1). Finally, module 15—by far the largest module, containing almost 16% of red oak's possible genes—is negatively associated with dormancy break (Figure 4). This module contains many orthologs, including several that were significant in a *Populus trichocarpa* GWAS for budflush and budbreak, as well as photoreceptors and components of the circadian clock (Table S1).

Looking more closely at the orthologs of EBB1, EBB3, CYCD 3.1, and SVL, we found that while they were all differentially expressed across our treatments, only orthologs of CYCD 3.1 clustered into any of the modules (Module 15). The rest were considered unassigned, meaning that their gene expression patterns were not similar enough to other genes to assign them to a module. We found that dormancy break was associated with an increase in expression across all orthologs, while transcript abundance of EBB1 and SVL began to lower after stage 1 (Figure 6).

### 3.3 What Genes Are Involved in Plasticity to Photoperiod and Temperature? As in Dormancy Break, Are There Orthologous Genes Annotated in Previous Studies Involved in These Responses?

Temperature was significantly associated with four modules—Modules 1, 3, 4, and 18 (Figure 4). Modules 1, 3, and 4 were discussed previously as they are also significantly associated with dormancy break. However, these modules are negatively correlated with temperature, while they are positively correlated with dormancy break (Figure 4). Module 18 is weakly, but significantly, correlated with temperature differences and contains an ortholog of a gene found in *Populus trichocarpa* via GWAS to be associated with bud flush (Table S1).

Photoperiod shares a negative correlation with modules 1, 3, and 4, as for temperature, but is also positively correlated with module 15. This pattern is the inverse of dormancy break, which was correlated with the same modules, but in the opposite direction. Photoperiod is also the only trait significantly correlated with the large set of genes (12,280) which were not able to be assigned to modules. These include orthologs like EBB1 and EBB3 that were found to be central to budburst behavior in *Populus* (Langfelder and Horvath 2008; Laube et al. 2014; Li and Dewey 2011). Notably, while we uncovered a significant interaction in the phenotypic data between photoperiod and temperature, this interaction did not significantly correlate with any of the modules.

## 4 Discussion

Here we used a growth chamber experiment of excised red oak ( *Quercus rubra* ) branches to uncover the genes associated with the timing of leaf development in spring. We importantly structured our study to look at how two genotypes behaved across a factorial of two spring warming by short/long day photoperiod treatments (four total treatments). We found significant phenotypic differences between genotypes and across treatments, and these corresponded with significant changes in gene expression. Our study is hopefully the first of many in attempting to untangle the vital trait of leaf development phenology in temperate trees using an ecologically important species. Our work is also unique in its approach, using cuttings as genetic replicates from mature trees rather than half‐sibling seedlings, as has traditionally been the approach for example, (Soneson et al. 2015).

### 4.1 Temperature, Photoperiod, and Genetic Background Influence the Phenotype of Leaf‐Out Timing in Red Oak

As expected, we found significant differences among genotypes and treatments in their leaf development timing (Figures 2 and 3). There is ample data that demonstrate that spring leaf phenology in temperate trees is both plastic in response to longer days and warmer temperatures, for example, (Evans et al. 2014; Finzi et al. 2020; Tepperman et al. 2006), but also highly heritable and locally adapted, for example, (Cavender‐Bares et al. 2015). Notably, we found that while photoperiod was a significant driver of leaf‐out variation, it still had a much smaller effect size than temperature, which is contrary to some previous work that suggests red oak is particularly sensitive to photoperiod changes, for example, (Blumstein et al. 2024). Altogether, our findings that leaf phenology in these two oak trees is both heritable and sensitive to environmental variation is confirmation that our study design identified biologically meaningful processes.

Trees are long‐lived, slow growing, and often very large, making controlled experiments to ascertain their response to environmental change extremely challenging. Past studies have approached this problem by using seedlings or saplings in growth chambers or experimental manipulations to study the impact of the environment on leaf development. However, there are two issues with this approach. The first is that for some species, oak included, it is difficult to make genetic clones via cuttings. Thus, seedlings or saplings are half‐siblings at best for these species, making it difficult to isolate variation caused by genes versus the environment. Our finding that genotype was the primary driver of variation in gene expression on MDS 1 here highlights the possible confounding effect of genetic diversity in such studies. The second challenge with using younger material is that phenology has an ontogenetic component, where younger tissues (seedlings/saplings) tend to leaf out earlier than their mature counterparts, for example, (Tsarouhas et al. 2003). The use of cuttings from mature trees, as we have done here, has been compared to the phenology of the original tree in situ. In one study, Vitasse and Basler found that twigs excised from mature trees closely followed the phenological timing of the parent tree in situ and more so than seedlings and saplings (Ueno et al. 2013). In addition, Laube et al. placed excised cuttings of 36 different species in growth chambers and found the rank order of leaf out matched the in situ parent trees (Chuine 2000). Together this past work and our results suggest that our use of excised cuttings accurately captures variation of mature trees in the wild and allows us to clonally replicate genotypes across treatments, which is essential for isolating drivers of phenotypic and expression variation.

### 4.2 Gene Expression Underlying Dormancy Break

Through a network analysis, we found several gene modules that were significantly associated with dormancy break across all treatments, highlighting possible candidates for further investigation for their role in leaf development. Within these modules, we found several orthologs to other variants identified in *Populus* species as related to bud flush or photoreception (Table S1). These findings are a critical first step to understanding the mechanisms underlying phenology in temperate trees. Given the challenges of working with tree species and the (until recently) high cost of transcriptome sequencing, few functional genomic studies have been conducted on nonmodel plants. In tree species outside of the model *Populus*, to our knowledge, there are only a handful of studies that have examined differential expression across leaf development stages (Martin 2011; Soneson et al. 2015; Vimont et al. 2019) and none that took our approach of using excised branches in chambers.

To date, most work on connecting genotypes to phenotypes in tree species has occurred in the model genus *Populus*. For example, a series of studies over the last decade in the cross *Populus tremula × Populus alba* have identified the genes EBB1, EBB3, CYCD 3.1, and SVL as important to the dormancy break pathway (Stage 0–1 in our study) (Langfelder and Horvath 2008; Laube et al. 2014). In our work, we found orthologs for these genes in red oak and found that they were likewise differentially expressed as a function of genotype, growth temperature, and growth photoperiod. However, more work needs to be done to confirm whether these genes play similar roles in oak as they do in populus. For example, we find that these candidate genes are differentially expressed in the expected ways given the pathway identified in the populus cross (Figure 6A). However, only one of the orthologs was assigned to a gene cluster in our analysis. This does not mean that these orthologs are not important or not related to dormancy break, but is more a reflection that our dataset is quite large and complex. In total, we identified transcripts from over 30,000 genes out of a possible 47,000 in our original dataset. Our results suggest what others have before (Kuznetsova et al. 2017; Singh et al. 2018), that phenological timing is likely a highly polygenic and complex trait.

### 4.3 Gene Expression Underlying Plasticity to Photoperiod and Temperature

In our co‐expression analysis, we uncovered four modules associated with temperature and photoperiod each. Notably, the two treatment factors shared three of the four modules, and the direction of the correlation was the same in all cases (Figure 4). These three core modules are also shared with dormancy break, suggesting that they may play a significant role in leaf development. While there are a few GWAS bud flush orthologs in our dataset and one for a photoreceptor, more work should be done to understand their core functions.

Where the two treatments differ also highlights interesting areas for further study. Photoperiod and dormancy are additionally associated with module 15, while temperature is not. Module 15 is by far the largest in our analysis with 7543 genes with some interesting orthologs within it. Notably, module 15 contains many orthologs for photoreceptors and several for genes involved in the circadian clock, as well as many budflush‐associated loci from earlier GWAS (Table S1). In particular, module 15 contains orthologs for phytochromes and related proteins initially discovered in *Arabidopsis* (PHYA, PHYB1, PHYB2, HY2.1, HY1.2). Recent advances have demonstrated that phytochromes may play a much wider role in environmental sensing beyond light (Vitasse 2013), signaling for germination (Vitasse and Basler 2014), transition from underground elongation to aboveground development (Willis et al. 2010; Xu et al. 2015), thermosensing (Yamaguchi et al. 1998; Yordanov et al. 2014), and even inducing gravitropism (Zohner et al. 2016). These findings suggest that module 15's association with both dormancy and photoperiod could be the result of phytochrome flexibility or shared architectures.

Finally, although we did expressly contrast the two genotypes used herein, we did find four modules significantly associated with differential patterns of expression between the two. Interestingly, except for module 18, none of these correlated with any other experimental factor. The modules (2, 11, and 17) did not have any orthologs to leaf development pulled from other studies within them either. This suggests these genes are truly unique to differences between genotypes and unrelated to the experimental design of our study.

## Disclosure

Benefits generated: This research creates benefits due to all data and results being shared and made available on NCBI SAR.

## Conflicts of Interest

The authors declare no conflicts of interest.

## Supporting information


**Table S1:** pei370078‐sup‐0001‐TableS1.docx.

## Data Availability

All raw data from this paper is available on NCBI SAR at BioProject ID: PRJNA1219517 located at http://www.ncbi.nlm.nih.gov/bioproject/1219517.
