# Supplementary material for: Gene Regulatory Changes Associated With Phenological Transitions in an Ecologically Significant Tree Species
Source: Plant Environ Interact. 2025 Aug 4;6(4):e70078. doi: 10.1002/pei3.70078 (PMC12320121; doi:10.1002/pei3.70078)
Supplement: Supplementary file 1 — Table S1: pei370078‐sup‐0001‐TableS1.docx. [file PEI3-6-e70078-s001.docx]

**Supplemental Tables & Figures**

S1. Table of variants associated wtih budflush in other tree species drawn from the literature, the associated Q. rubra ortholog, and which module the orthologs were found in.

| Module | Hub Score | *Q. rubra* peptide | E-Value | Citation | Gene Name | Notes |
| --- | --- | --- | --- | --- | --- | --- |
| ME1 | 0.148 | Qurub.01G011100.2 | 1.44E-102 | ^57^ | . | GWAS budflush |
| ME1 | 0.632 | Qurub.01G070200.1 | 0 | Evans et al. 2014 | . | GWAS budflush |
| ME1 | 0.148 | Qurub.01G011100.2 | 1.44E-102 | ^58^ | PIF31 | Photoreceptors |
| ME10 | 0.01 | Qurub.01G069800.1 | 0 | Evans et al. 2014 | . | GWAS budflush |
| ME15 | 0.005 | Qurub.02G002400.1 | 0 | Keller et al. 2012 | ABI1B | ABA Sensitive |
| ME15 | 0.005 | Qurub.02G002400.1 | 0 | Keller et al. 2012 | ABI1D | ABA Sensitive |
| ME15 | 0.016 | Qurub.02G022800.1 | 1.41E-130 | ^59^ | CYCD3.1 | Downstream Targ. |
| ME15 | 0 | Qurub.11G098900.1 | 9.32E-110 | Azeez et al. 2021 | CYCD3.1 | Downstream Targ. |
| ME15 | 0.021 | Qurub.05G066400.1 | 2.26E-140 | Keller et al. 2012 | EBS | Downstream Targ. |
| ME15 | 0 | Qurub.06G189600.1 | 0 | Keller et al. 2012 | LFY | Downstream Targ. |
| ME15 | 0 | Qurub.12G109100.5 | 0 | ^60^ | . | GWAS bud break, LMA |
| ME15 | 0 | Qurub.01G009500.1 | 0 | Evans et al. 2014 | . | GWAS budflush |
| ME15 | 0 | Qurub.01G011500.1 | 5.34E-15 | Evans et al. 2014 | . | GWAS budflush |
| ME15 | 0 | Qurub.01G011600.1 | 0 | Evans et al. 2014 | . | GWAS budflush |
| ME15 | 0 | Qurub.01G018400.1 | 0 | Evans et al. 2014 | . | GWAS budflush |
| ME15 | 0.001 | Qurub.02G015600.1 | 0 | Evans et al. 2014 | . | GWAS budflush |
| ME15 | 0.108 | Qurub.02G339000.1 | 0 | Evans et al. 2014 | . | GWAS budflush |
| ME15 | 0.108 | Qurub.02G339000.1 | 0 | Evans et al. 2014 | . | GWAS budflush |
| ME15 | 0 | Qurub.03G079200.1 | 1.87E-61 | Evans et al. 2014 | . | GWAS budflush |
| ME15 | 0 | Qurub.03G192000.1 | 0 | Evans et al. 2014 | . | GWAS budflush |
| ME15 | 0 | Qurub.04G006500.1 | 0 | Evans et al. 2014 | . | GWAS budflush |
| ME15 | 0.005 | Qurub.05G252100.1 | 0 | Evans et al. 2014 | . | GWAS budflush |
| ME15 | 0 | Qurub.05G253200.1 | 4.26E-49 | Evans et al. 2014 | . | GWAS budflush |
| ME15 | 0 | Qurub.06G029400.1 | 2.61E-57 | Evans et al. 2014 | . | GWAS budflush |
| ME15 | 0.008 | Qurub.06G029600.1 | 2.87E-52 | Evans et al. 2014 | . | GWAS budflush |
| ME15 | 0 | Qurub.06G128700.1 | 0 | Evans et al. 2014 | . | GWAS budflush |
| ME15 | 0.001 | Qurub.06G129200.1 | 0 | Evans et al. 2014 | . | GWAS budflush |
| ME15 | 0.002 | Qurub.06G213200.1 | 6.10E-53 | Evans et al. 2014 | . | GWAS budflush |
| ME15 | 0.009 | Qurub.06G251300.2 | 4.63E-49 | Evans et al. 2014 | . | GWAS budflush |
| ME15 | 0.002 | Qurub.07G082900.1 | 0 | Evans et al. 2014 | . | GWAS budflush |
| ME15 | 0 | Qurub.07G090900.1 | 1.01E-177 | Evans et al. 2014 | . | GWAS budflush |
| ME15 | 0.019 | Qurub.07G102400.1 | 0 | Evans et al. 2014 | . | GWAS budflush |
| ME15 | 0.023 | Qurub.07G209600.1 | 1.15E-96 | Evans et al. 2014 | . | GWAS budflush |
| ME15 | 0 | Qurub.08G291500.1 | 0 | Evans et al. 2014 | . | GWAS budflush |
| ME15 | 0.212 | Qurub.09G047800.1 | 0 | Evans et al. 2014 | . | GWAS budflush |
| ME15 | 0.212 | Qurub.09G047800.1 | 0 | Evans et al. 2014 | . | GWAS budflush |
| ME15 | 0.077 | Qurub.09G115800.1 | 3.59E-132 | Evans et al. 2014 | . | GWAS budflush |
| ME15 | 0 | Qurub.09G153800.2 | 0 | Evans et al. 2014 | . | GWAS budflush |
| ME15 | 0.01 | Qurub.09G157900.1 | 0 | Evans et al. 2014 | . | GWAS budflush |
| ME15 | 0.064 | Qurub.10G061500.2 | 0 | Evans et al. 2014 | . | GWAS budflush |
| ME15 | 0.012 | Qurub.10G158500.1 | 7.36E-101 | Evans et al. 2014 | . | GWAS budflush |
| ME15 | 0.001 | Qurub.10G158600.2 | 5.23E-09 | Evans et al. 2014 | . | GWAS budflush |
| ME15 | 0.042 | Qurub.10G162400.1 | 0 | Evans et al. 2014 | . | GWAS budflush |
| ME15 | 0 | Qurub.10G193100.1 | 0 | Evans et al. 2014 | . | GWAS budflush |
| ME15 | 0.002 | Qurub.11G055600.1 | 0 | Evans et al. 2014 | . | GWAS budflush |
| ME15 | 0.021 | Qurub.12G006800.1 | 8.28E-26 | Evans et al. 2014 | . | GWAS budflush |
| ME15 | 0 | Qurub.12G100300.2 | 1.93E-06 | Evans et al. 2014 | . | GWAS budflush |
| ME15 | 0 | Qurub.12G170000.1 | 2.41E-168 | Evans et al. 2014 | . | GWAS budflush |
| ME15 | 0.001 | Qurub.04G079000.8 | 6.49E-172 | Keller et al. 2012 | CKB34 | Peri Circ Clock |
| ME15 | 0.084 | Qurub.10G236100.1 | 1.43E-104 | Keller et al. 2012 | ELF3 | Peri Circ Clock |
| ME15 | 0.029 | Qurub.01G127800.1 | 1.37E-95 | Keller et al. 2012 | HY1.2 | Photoreceptors |
| ME15 | 0.029 | Qurub.01G127800.1 | 5.01E-95 | Keller et al. 2012 | HY2.1 | Photoreceptors |
| ME15 | 0.029 | Qurub.01G127800.1 | 3.18E-94 | Keller et al. 2012 | HY2.1 | Photoreceptors |
| ME15 | 0.029 | Qurub.01G127800.1 | 6.46E-96 | Keller et al. 2012 | HY2.1 | Photoreceptors |
| ME15 | 0 | Qurub.06G189600.1 | 0 | Keller et al. 2012 | PHYA | Photoreceptors |
| ME15 | 0 | Qurub.06G189600.1 | 0 | Keller et al. 2012 | PHYB1 | Photoreceptors |
| ME15 | 0 | Qurub.03G002600.1 | 0 | Keller et al. 2012 | PHYB2 | Photoreceptors |
| ME16 | 0.002 | Qurub.02G147400.1 | 0 | McKown et al. 2014 | . | GWAS bud break, LMA |
| ME16 | 0.843 | Qurub.12G064300.1 | 0 | Evans et al. 2014 | . | GWAS budflush |
| ME17 | 0.001 | Qurub.08G215400.1 | 0 | Evans et al. 2014 | . | GWAS budflush |
| ME18 | 0.415 | Qurub.07G103100.1 | 0 | Evans et al. 2014 | . | GWAS budflush |
| ME4 | 0.065 | Qurub.10G233000.1 | 0 | Keller et al. 2012 | HY2.2 | Photoreceptors |
| ME5 | 0.448 | Qurub.02G134900.1 | 0 | Evans et al. 2014 | . | GWAS budflush |
| ME7 | 0.99 | Qurub.09G189600.1 | 0 | Keller et al. 2012 | ABI3 | ABA Sensitive |
| ME7 | 0.687 | Qurub.05G022100.1 | 0 | Evans et al. 2014 | . | GWAS budflush |
| ME7 | 0.706 | Qurub.05G049500.1 | 1.40E-174 | Evans et al. 2014 | . | GWAS budflush |
| ME7 | 0.998 | Qurub.10G053900.1 | 4.54E-118 | Evans et al. 2014 | . | GWAS budflush |
| ME7 | 0.978 | Qurub.11G174400.1 | 0 | Evans et al. 2014 | . | GWAS budflush |
| ME8 | 0.06 | Qurub.12G064400.1 | 0 | Evans et al. 2014 | . | GWAS budflush |

a
